# A frailty index based on routine laboratory data predicts increased risk of mortality in Chinese community-dwelling adults aged over 55 years: a five-year prospective study

**DOI:** 10.1186/s12877-022-03374-z

**Published:** 2022-08-17

**Authors:** Chunxiu Wang, Xianghua Fang, Zhe Tang, Yang Hua, Zhongying Zhang, Xiang Gu, Beibei Liu, Xunming Ji

**Affiliations:** 1 Department of Evidence-Based Medicine, Xuanwu Hospital, Capital Medical University, No 45 Changchun Street, Xicheng district, Beijing, China; 2 Department of Vascular Ultrasonography, Xuanwu Hospital, Capital Medical University, Beijing, China; 3 Geriatric Department, Xuanwu Hospital, Capital Medical University, Beijing, China; 4 Geriatric Department, Affiliated Beijing Friendship Hospital, Capital Medical University, Beijing, China; 5 Department of Neurological Surgery, Xuanwu Hospital, Capital Medical University, No 45 Changchun Street, Xicheng district, Beijing, China

**Keywords:** Frailty, Older adults, Mortality, Laboratory data, Aging

## Abstract

**Background:**

Frailty can be operationalized based on the accumulation of deficits using a frailty index (FI) and is associated with an increased risk of adverse health outcomes. Here, we aim to compare validity of a FI from laboratory data with that of the common clinical FI for prediction of mortality in adults aged 55 + years, also examine whether combined FI could improve identification of adults aged 55 + years at increased risk of death.

**Methods:**

Data for this analysis were obtained from the Beijing Longitudinal Study of Aging that involved 1,257 community-dwelling Chinese people, aged 55 + years at baseline. The main outcome measure was 5-year mortality. An FI-self-report based on 30 self-reported health-related data was constructed. An FI-lab was developed using laboratory data, in addition to pulse, systolic and diastolic blood pressure, pulse pressure, body mass index (BMI) and waist. A combined FI comprised all items from each FI. Kaplan–Meier survival curve and Cox proportional hazards models were performed to evaluate the risk of each FI on death. The area under receiver operating characteristic(ROC) curves were used to compare the discriminative performance of each FI.

**Results:**

Of 1257 participants, 155 died and 156 lost at the end of the 5-year follow-up. The mean FI-self-report score was 0.11 ± 0.10, the FI-lab score was 0.33 ± 0.14 and FI-combined score was 0.19 ± 0.09. Higher frailty level defined by each FI was associated with higher risk of death. After adjustment for age and sex, Cox proportional hazards models showed that the higher scores of frailty were associated with a higher risk of mortality for each FI, the hazard ratios for the FI-self-report and FI-lab and FI-combined were 1.04 (1.03 to 1.05) and 1.02 (1.01 to 1.03) and 1.05 (1.04 to 1.07), respectively. The areas under the ROC curve were 0.79 (0.77–0.82) for the FI-self-report, 0.77(0.75–0.80) for the FI-lab and 0.81(0.78–0.82) for FI-combined.

**Conclusions:**

A FI from laboratory data can stratify older adults at increased risk of death alone and in combination with FI based on self-report data. Assessment in clinical settings of creating an FI using routine collected laboratory data needs to be further developed.

**Supplementary Information:**

The online version contains supplementary material available at 10.1186/s12877-022-03374-z.

## Introduction

Frailty represents an increased multisystem decline and an increased vulnerability with age. Individuals with a higher level of frailty are more likely to have poor health outcomes, thus leading to a higher risk of institutionalization and death [1–5]. A more controversial issue is how best to standardize and operationalize frailty in routine care and clinical settings, as well as for research purposes [5, 6]. Among these considerations, a common definition recognizes frailty as the accumulation of deficits across various domains (e.g., symptoms, signs, laboratory abnormalities, diseases and disabilities) that are combined into a frailty index score, which reflects the proportion of accumulated health deficits that are present in a person. The ability to measure the degree of frailty of a specific individual is useful in health and clinical care settings. Early identification can help to develop interventions or to predict patients’ risks of future institutional care and death. The characteristics of FI increasing with age and having a close association with the risk of death have been demonstrated by multiple data sources, including data from our previous studies [3, 4, 7].

Commonly, people accumulate more health deficits as they age, and the basic idea of FI is almost universally agreed upon, which states that the more health deficits an individual accumulates, the greater the individuals are at risk of possessing adverse health outcomes. The FI-Lab is composed entirely of routine physical assessment and laboratory test results and is used to study frailty in regards to variable deficit accumulation. In contrast to single measurements of physical capability, the manner in which laboratory tests correspond to major and minor deficits is not clear, and further investigations are needed.

A growing body of research literature has focused on an understanding of the mechanisms that are hypothesized to link frailty with age, particularly concerning how subclinical frailty based on subclinical deficits (e.g., laboratory test abnormalities, aging biomarkers and atherosclerosis) advances the early manifestations of clinically visible frailty [8, 9]. Recently, a study using Canadian data reported that the gathered subclinical deficits (e.g., laboratory test abnormalities) were significantly associated with mortality risks [10], and a consistent finding from European data demonstrated that increasing FI-Lab scores have been associated with a higher risk of death [11]. These studies suggested that the addition of laboratory test data is important for a better understanding of the associations between laboratory test abnormalities and frailty, as well as the possible mechanisms that are involved.

We previously reported that the standard FI based on cumulative self-reported health deficits can predict a range of other health outcomes, including death, dementia, falls, fractures, disability and transition probability, which were independent of a number of covariates in multivariable models [4, 12–17]. However, these studies have primarily examined the standard FI (e.,g., FI based on self-reported data) on the risk of death, but with limited evidence on whether an aggregation of the presence of laboratory test abnormalities may contribute to the risk of mortality in Chinese adults aged over 55 years. Additionally, a previous study demonstrated that the 68-item FI-combined exhibited a higher hazard ratio for death than did the 36-item FI-Self-report and 32-item FI-Lab [10, 11]. Similarly, we questioned whether the combination of laboratory values with self-reported items increased the association of the FI with higher mortality rates in Chinese adults aged over 55 years, in order to determine if the addition of the FI-lab to a frailty screening measure may strengthen its predictive ability. Thus, the goal of the current study was to compare the FI-lab based on routine laboratory data to a FI-self-report; in addition, we examined whether the combination of the FI-lab with the FI-self-report increased the predictive ability of the frailty index in relation to death in a cohort of 1,257 community-dwelling Chinese adults aged over 55 years.

## Methods

### Study setting and participants

This was a secondary analysis of the Beijing Longitudinal Study of Aging (BLSA), which is a representative cohort survey of community-dwelling Chinese people aged 55 years and older at baseline (response rate: 91.2%). As described in detail in previous studies [7, 14, 15, 18], the present study is based on the data of the 2009 survey. All of the participants completed a self-reported standardized health questionnaire based on face-to-face interviews by trained interviewers, and the collected information contained demographic and socioeconomic characteristics, physical and cognitive function, psychological health, lifestyle and utilized health services. If available, medical records were used to verify the presence of a disease. In addition, each participant was invited to undergo physical examinations and blood tests and to be examined via carotid artery B-mode ultrasonography in the 2009 survey, and 1,257 individuals completed these clinical examinations. The survival data at the five-year follow-up (in 2014) were determined via interviews with family members or neighbors and verified via death certificates and/or local police register records. The study was approved by the Ethics Committee of Xuanwu Hospital, Capital Medical University, Beijing, China. Written informed consent was obtained from each participant in the Beijing Longitudinal Study of Aging.

### Construction of the FI

The FI was constructed following a standard procedure that has been described in our earlier reports by our group [7, 14]. In particular, a FI self-report was created by using 30 variables selected as health deficits; they were selected if they are associated with health status, accumulate with age, do not saturate too early, have a prevalence greater than 1% and have < 5% missing values. The variables that comprised the FI encompassed a range of health problems, including diseases (*n* = 9), symptoms (*n* = 6), living disabilities (*n* = 6), psychological problems (*n* = 2), Romberg Test (*n* = 4) [19], physical performance, GDS score and MMSE score (Supplementary Table 1). For the binary variables, the presence of a deficit was coded as “1”, and its absence was coded as ‘0’ (*n* = 21). For each three-level variable (*n* = 9), 0.5 was used to represent an intermediate response level. The MMSE was coded as 0 (MMSE > 23), 0.5 (MMSE = 15–23) and 1 (MMSE ≤ 14), whereas the GDS was recoded as 0 (GDS < 11) and 1 (GDS ≥ 11). Second, the FI-lab identified 15 deficits based on 9 laboratory tests, in addition to pulse, systolic and diastolic blood pressure, pulse pressure, BMI and waist circumference (Table 1). A normal reference range was used to code each deficit [20–23], wherein ‘0’ indicated that values were within the normal range and ‘1’ indicated that values were outside of the reference range. Finally, we combined the two FIs to build a 45-item FI (FI-combined). Therefore, each FI was defined as the number of deficits of an individual divided by the total number of considered deficits, with the FI ranging from a theoretical minimum of 0 (no deficits present) to a possible maximum of 1.0 (all deficits present). No individual had > 5% missing deficit items.

**Table 1:** Variables and coding used to construct the 15-item FI-lab and their present (%)

Variables	Cut-off (yes = 1, no = 0)	Present (%)
SBP, mmHg	< 90 or ≥ 140	43.8
DBP, mmHg	< 60 or ≥ 90	18.0
Pulse pressure, mm Hg	> 65 or < 30	37.9
Pulse, bp/min	< 60 or > 100	7.9
BMI, kg/m^2^	< 18.5 or ≥ 25	41.0
CR, umol/L	< 53 or ≥ 106	41.8
HCY, mmol/L	> 15	60.5
FBG, mmol/L	< 3.9 or ≥ 6.1	19.3
hsCRP, mmol/L	> 3	0.9
UA, umol/L	male < 149 or ≥ 420, female < 89 or ≥ 360	31.3
LDL-C, mmol/L	≥ 4.16	6.4
HDL-C, mmol/L	< 1.03	24.1
TC, mmol/L	< 3.9 or > 6.5	30.2
TG, mmol/L	≥ 2.26	15.0
Waist, cm	Male ≥ 90, female ≥ 85	63.5

*Abbreviations*: *BMI* Body mass index, *DBP* Diastolic blood pressure, *DM* Diabetes mellitus, *FBG* Fast blood glucose, *HDL-C* High-density lipoprotein cholesterol, *hsCRP* High-sensitivity C-reactive protein, *HCY* Homocysteine, *HT* Hypertension, *LDL-C* Low-density lipoprotein cholesterol, *SBP* Systolic blood pressure, *TC* Total cholesterol, *TG* Triglyceride, *UA* Uric acid, *CR* Serum creatinine

### Statistical analysis

All of the continuous variables are described with means and standard deviations, whereas the categorical variables are described with percentages. Baseline characteristics were compared by using analysis of variance (ANOVA) for the continuous variables and the chi-square test for the categorical variables. A Pearson correlation coefficient was used to examine the relationship between FI-self-report and FI-lab. We multiplied the index by 100 for the measurement of the deficits factor so that the change in risk could be observed with each percent increment of the FI [17]. The Kaplan‒Meier survival curves demonstrated differences in mortality rate by four frailty grades for each FI. Cox proportional hazard models were applied to explore the association between the FI and the risk of mortality in the full sample, after which they were stratified by age (age 65^+^ years), adjusted for age and sex. The performance of each FI in classifying individuals who died and who survived was assessed based on receiver operating characteristic (ROC) curves and the evaluation of the area under receiver operating characteristic curves (AUCs). We also used net reclassification index (NRI) to compare the predictive performance between competing models(i.e., FI-combined versus FI-self-report, FI-self-report versus FI-lab). All of the data analyses were conducted by using SPSS version 22.0, and graphs were created by using MedCalc 15.2. The statistical significance level was set at *p* < 0.05.

## Results

A total of 1,257 participants in the Beijing Longitudinal Aging Study were analyzed (707 women, 47.5%; mean age: 69.2 ± 8.1 years, range: 55 to 97 years). Table 2 summarizes the demographic characteristics of the study participants subdivided by grades of frailty for both the FI-self-report and the FI-lab. Of these study participants, 66.3% lived in rural areas, 31.8% completed at least 9 years of schooling and 30.8% and 30.5% were current smokers and current drinkers, respectively. Within the 5-year follow-up period, 155 (12.3%) people died (Supplementary Fig. 1). The mean FI-self-report score was 0.11 ± 0.10 (range: 0.00–0.67), which was lower than the FI-Lab (0.33 ± 0.14; range: 0.00–0.76) and the FI-combined (0.19 ± 0.09; range: 0.00–0.62). The 99th percentile scores for all of the frailty indices were below 0.7 (FI-self-report: 0.52; FI-lab: 0.68; FI-combined: 0.49). In addition, the Pearson correlation coefficient between the FI-Lab and the FI-Self-report was 0.23 (*p* < 0.001).

**Table 2:** Baseline demographic characteristics and mortality by grades of frailty

**Baseline characteristic**	**Total** ***N* = (1257)**		**Grades of the FI**		
		** < 0.20**	**0.20–0.29**	**0.30–0.39**	** > 0.40**
**FI-self-report**		*n* = 1066	*n* = 116	*n* = 38	*n* = 37
Mean age, years (± SD)	69.2 ± 8.1	68.1 ± 7.7	73.3 ± 7.4	76.8 ± 5.9	78.7 ± 9.1
Mean FI-self-report (± SD)	0.11 ± 0.10	0.08 ± 0.05	0.24 ± 0.03	0.35 ± 0.03	0.49 ± 0.07
Women, n (%)	707 (56.2)	572 (53.6)	85 (73.3)	23 (62.2)	27 (73.0)
Rural dwelling, n (%)	834 (66.3)	684 (64.1)	88 (75.9)	32 (86.5)	30 (81.1)
9^+^ years education, n (%)	395 (31.8)	369 (34.9)	20 (17.5)	2 (5.7)	4 (10.8)
Smoking, n (%)	386 (30.8)	344 (32.3)	27 (23.3)	10 (27.0)	5 (13.9)
Drinking, n (%)	384 (30.5)	342 (32.1)	26 (22.4)	11 (29.7)	5 (13.9)
Mortality, n (%)	155 (12.3)	90 (8.4)	24 (29.7)	19 (51.4)	22 (59.5)
**FI-lab**		** < 0.20**	**0.20–0.29**	**0.30–0.39**	** > 0.40**
		*n* = 199	*n* = 362	*n* = 221	*n* = 475
Mean age, years (± SD)	69.2 ± 8.1	66.1 ± 7.6	68.6 ± 8.2	69.8 ± 8.2	70.6 ± 7.5
Mean FI-lab (± SD)	0.33 ± 0.14	0.10 ± 0.04	0.24 ± 0.03	0.34 ± 0.01	0.49 ± 0.08
Women, n (%)	707 (56.2)	108 (54.3)	213 (58.8)	127 (57.5)	259 (54.5)
Rural dwelling, n (%)	834 (66.3)	130 (65.3)	241 (66.6)	151 (68.3)	312 (65.7)
9^+^ years education, n (%)	395 (31.8)	80 (40.8)	117 (32.3)	68 (30.8)	130 (27.3)
Smoking, n (%)	386 (30.8)	58 (29.1)	110 (30.4)	68 (30.8)	150 (31.6)
Drinking, n (%)	384 (30.5)	60 (29.9)	110 (30.5)	66 (29.9)	148 (31.1)
Mortality, n (%)	155 (12.3)	13 (6.5)	35 (9.7)	30 (13.6)	77 (16.3)
**FI-combined**		** < 0.20**	**0.20–0.29**	**0.30–0.39**	** > 0.40**
		*n* = 770	*n* = 338	*n* = 110	*n* = 39
Mean age, years (± SD)	69.2 ± 8.1	67.3 ± 7.5	70.8 ± 8.0	74.2 ± 7.2	76.8 ± 8.4
Mean FI-combined (± SD)	0.19 ± 0.09	0.13 ± 0.04	0.24 ± 0.03	0.34 ± 0.03	0.46 ± 0.05
Women, n (%)	707 (56.2)	410 (53.2)	191 (56.5)	77 (70.0)	29 (74.4)
Rural dwelling, n (%)	834 (66.3)	488 (63.4)	229 (67.8)	85 (77.3)	32 (82.1)
9^+^ years education, n (%)	395 (31.8)	291 (37.8)	82 (24.3)	17 (15.5)	5 (12.8)
Smoking, n (%)	386 (30.8)	244 (31.6)	103 (30.5)	32 (29.1)	7 (17.9)
Drinking, n (%)	384 (30.6)	253 (32.9)	96 (28.4)	26 (23.6)	9 (23.0)
Mortality, n (%)	155 (12.3)	56 (7.3)	44 (13.0)	32 (29.1)	23 (59.0)

*Abbreviations*: *FI* Frailty index, *SD* Standard deviation

With each FI, as the baseline frailty scores increased, the mean age also increased, and the trend was present across all three age groups (Tables 2 and 3). The mean FI-lab score was higher than the FI-self-report score in the group with the lowest degree of frailty. Additionally, mean FI-self-report scores increased from 0.08 ± 0.05 in the least frail group to 0.49 ± 0.07 in the frailest group (Table 2). The average FI-lab values also increased from 0.10 ± 0.04 in the group with the lowest scores to 0.49 ± 0.08 in the group with the highest degree of frailty (Table 2). Similarly, the mean combined FI scores increased from 0.13 ± 0.04 in the group with the lowest scores to 0.46 ± 0.05 in the group with the highest frailty scores (Table 2). Moreover, adults aged over 65 years had higher frailty levels than the full sample in the FI-self-report, FI-lab and FI-combined; however, the average FI values in those aged 65^+ ^years were similar to the full sample from the least frail group to the frailest group in the three FIs (Supplementary Table 2).

**Table 3:** Baseline gender and FIs and 5-year mortality by age-group

Baseline characteristic	Age-group (years)		
	55–64	65–74	> 75
	*n* = 414	*n* = 478	*n* = 365
Women, n (%)	242 (58.5)	270 (56.5)	195 (53.4)
Mortality, n (%)	14 (3.4)	43 (9.0)	98 (26.8)
Mean FI-self-report (± SD)	0.08 ± 0.07	0.10 ± 0.09	0.17 ± 0.13
Mean FI-lab (± SD)	0.28 ± 0.15	0.33 ± 0.15	0.35 ± 0.13
Mean FI-combined (± SD)	0.15 ± 0.07	0.19 ± 0.09	0.23 ± 0.10

*Abbreviations*: *FI* Frailty index

The participants with higher levels of frailty tended to be less educated, and this effect was similar across all three FIs, as well as in the full sample and for those aged over 65 years (Table 2, Supplementary Table 2). Of note, the proportion of women and rural dwelling people with low FI scores was much lower when frailty was stratified by FI-lab scores compared to the FI-self-report and the FI-combined; however, the pattern was reversed in less educated, smoking and drinking people in the full sample and those individuals aged over 65 years (*p* < 0.001) (Table 2, Supplementary Table 2).

The mean frailty level increased with age in all three FIs (Table 3). In the FI-self report, FI-lab and FI-combined, there was an increase in frailty across all three age groups (*p* < 0.001).

**Fig. 1:**
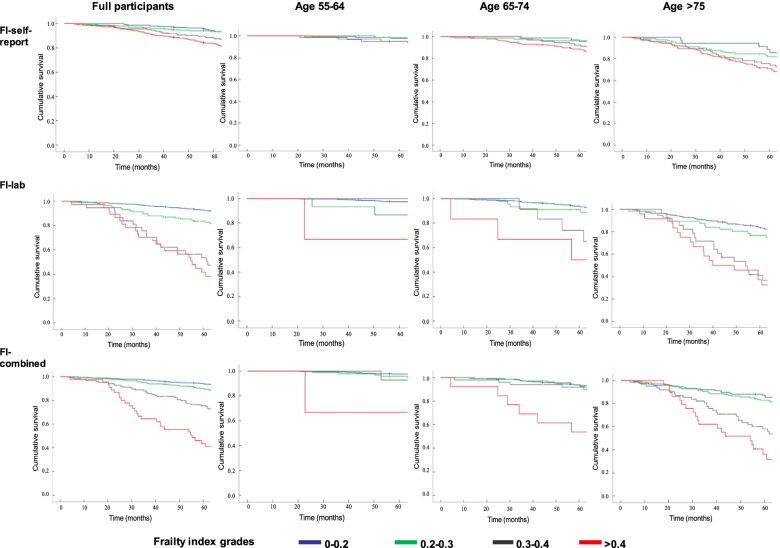
Kaplan–Meier curves for FI-self-report, FI-Lab and FI-Combined showing the relationship of frailty levels with time to death

Mortality rates absolutely increased as each FI score increased. In addition, participants with higher frailty had a much higher mortality than those individuals with the lowest frailty, regardless of which FI was used. This effect was more noticeable with the FI-self-report than with the FI-lab scores (Table 2), and mortality was also obviously increased with age in the three age groups (Table 3). In the proportional hazards analyses, adjusted for sex and age, the higher scores of frailty were also associated with a higher risk of mortality for each FI (Table 4). The risk of mortality for each 0.01 frailty score increase was higher for the FI-self-report than for the FI-lab and remained individually significant when both factors were included in the same model for death prediction. Furthermore, the hazard ratios for the FI-self-report, FI-lab and FI-combined were 1.04 (1.03 to 1.05), 1.02 (1.01 to 1.03) and 1.05 (1.04 to 1.07), respectively (Table 4). These findings were confirmed in the survival curve analysis. The Kaplan–Meier curves showed a significant separation for all FIs, with the FI-combined demonstrating the clearest separation, particularly in the over 75-year group by grades of frailty. Similar results were presented when stratifying by three age groups (Fig. 1).

**Table 4:** Comparison of FI-self-report vs FI-lab vs FI-combined for Prediction of 5-year mortality

	B	SE	Wald Statistic	HR(95%CI)^a^	AUC (95% CI)
**FI-self-report**					0.79^b^(0.77–0.82)
Age	0.08	0.01	53.88	1.09 (1.06–1.11)*	
Female	-0.56	0.17	11.35	0.57 (0.41–0.79)*	
FI-self-report	0.04	0.01	44.22	1.04 (1.03–1.05)*	
**FI-lab**					0.77^c^(0.75–0.80)
Age	0.11	0.01	107.21	1.12 (1.10–1.14)*	
Female	-0.34	0.16	4.35	0.72 (0.52–0.98)*	
FI-lab	0.02	0.01	9.54	1.02 (1.01–1.03)*	
**FI-combined**					0.81^d^(0.78–0.82)
Age	0.09	0.01	61.02	1.09 (1.07–1.12)*	
Female	-0.54	0.17	10.53	0.58 (0.42–0.81)*	
FI-combined	0.05	0.01	46.23	1.05 (1.04–1.07)*	
**FI-self-report & FI-lab**					
Age	0.08	0.01	53.12	1.09 (1.06–1.11)*	
Female	-0.56	0.17	11.41	0.57 (0.41–0.79)*	
FI-self-report	0.04	0.01	38.04	1.04 (1.03–1.05)*	
FI-lab	0.01	0.01	4.86	1.02 (1.00–1.03)*	

*Abbreviations*: *FI* Frailty index, *AUC* Area under the curve, *CI* Confidence interval, *SE* Standard error, *HR* Hazard ratio,*p<0.05 was considered  as significance

^a^ Adjusted for age and sex

^b^ *p* = .042 for comparison of FI-self-report vs FI-lab

^c^ *p* = .028 for comparison of FI-lab vs FI-combined

^d^ *p* = .049 for comparison of FI-self-report vs FI-combined

**Fig. 2:**
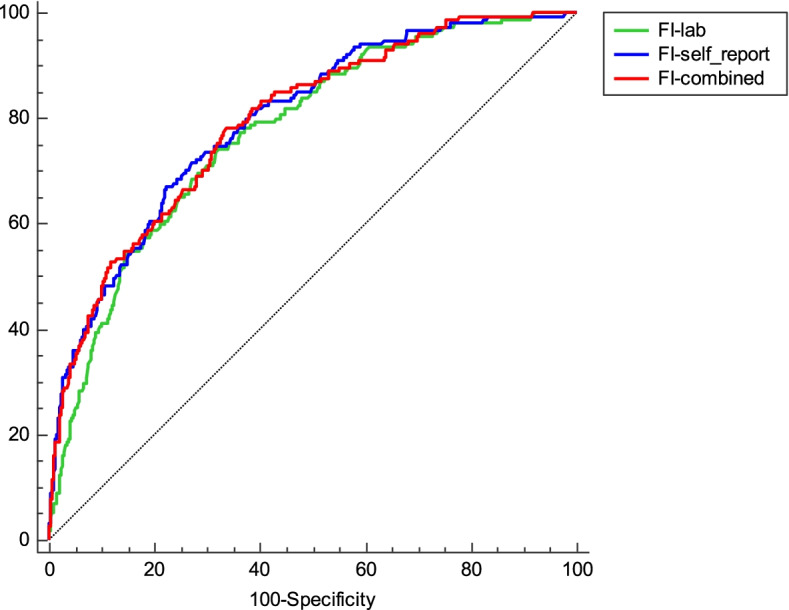
Receiver operating characteristic (ROC) curves showing the performance of the frailty index in predicting individuals who died

ROC curves demonstrated the acceptable discrimination performance of the frailty index for predicting the 5-year mortality rate. The discriminative ability for mortality did not exhibit a significant increase when the FI-self-report and FI-lab were combined. Moreover, there was no difference with the FI-self-report compared to those with the FI-combined in discriminating death (*p* = 0.669). The AUCs were 0.79 (0.77–0.82) for FI-self-report, 0.77 (0.75–0.80) for the FI-lab and 0.81 (0.78–0.82) for FI-combined (Fig. 2).

Furthermore, to explore the relationship between each FI and adverse health outcomes in older adults over the age of 65, we conducted a stratified analysis (Supplementary Table 3). When stratifying by age (individuals aged over 65 years), the results were similar to those of the full sample; specifically, the hazard ratios were 1.04 (FI-self-report), 1.02 (FI-lab) and 1.05 (FI-combined). All of the associations remained significant after adjusting for age and sex. The AUCs were 0.75 (0.72–0.78) for FI-self-report, 0.73 (0.70–0.76) for the FI-lab and 0.76 (0.72–0.78) for FI-combined (Supplementary Fig. 2). However, there was no difference in the multivariable models with the FI-self-report compared to those with the FI-combined in discriminating mortality (*p* = 0.786). FI-combined outperformed FI-self-report with significant prediction increment (NRI = 0.375, *p* < 0.05), compared with prediction using FI-self-report. No significant prediction increment (NRI = 0.026, *p* = 0.442) was observed between FI-self-report and the FI-lab.

## Discussion

In the context of previously reported results from the Beijing Longitudinal Study of Aging, a FI lab created from 9 laboratory tests, in addition to pulse, systolic and diastolic blood pressure, pulse pressure, BMI and waist circumference, had similar characteristics to the previously validated FI that was constructed from self-reported health items [7, 12]. Our study replicates prior findings from secondary analyses of similar datasets from Canada and Europe [10, 11]. As expected, both the mean FI-lab and the prevalence of high frailty levels were found to increase with age, and higher FI scores were associated with greater mortality [24, 25]. These associations were present across all three age groups (55–64 years, 65–74 years and > 75 years) in older people. Of note, the mean FI-lab score was higher than the mean FI-self-report score. Compared to the FI-self-report (and FI-combined), the FI-lab exhibited smaller hazard ratios associated with mortality, as has been previously reported in other settings [11, 26].

Herein, we demonstrated that the inclusion of more deficits in FI can enhance its prediction of mortality; specifically, the 45-item FI-combined exhibited higher hazard ratios than did the 30-item FI-self-report and the 15-item FI-lab. This implied that the quantity and/or the differential nature of the laboratory test items may provide more important information on mortality.

This was the first community-based cohort study in older Chinese adults to examine the properties and predictive value of FI based on laboratory data. The sample size was relatively large, with a comprehensive set of measurements, standard central laboratory testing and a long-term follow-up period. However, several potential limitations should be mentioned. First, the participants in the study well represented the local residents, and it was not a nationally representative sample. However, bias may have existed because 45.4% of the participants were not included in the analyses if they did not receive laboratory and ultrasonic assessments in the study. Thus, we compared the different characteristics of the entire cohort and the samples that were analyzed in this study, and there was no significant difference in the demographic data (except for age; those individuals without such measurements were older). Therefore, our results may slightly underestimate the relationship between FI and death. In addition, we compared the baseline demographic characteristics and health conditions of these people who were lost to follow-up with those individuals who survived, and the results of the analysis suggested small differences (Supplementary Table 4). Second, the current laboratory test only contained limited sendocrine, metabolic and inflammatory biomarkers, and other routine blood testing items were unavailable for analysis in the current study. It is not clear from prior studies whether the varieties and numbers of the abnormality items would add value to a FI based on laboratory data [27, 28]. This question needs to be further addressed. Third, to date, there have been few uniform normal reference ranges for abnormalities in blood testing that have been introduced in previous studies [10]. Indeed, future research on defining standard ranges on routinely collected laboratory data to a FI in clinical settings will be of particular interest.

In the current study, we investigated the predictive ability of the frailty indices in relation to death. With each version of the FI, higher FI scores were associated with a higher risk of mortality, as demonstrated in a multivariable model adjusted for age and sex. These findings suggested that the FI-lab provides a useful operationalization of frailty to use in routine care for identifying high-risk older adults, which was consistent with previous studies [10, 11]. Compared with the FI based on a geriatric assessment, the FI-Lab had a slightly lower ability to predict an increased risk of death in age-adjusted multivariable models. Regardless, it is vital to identify frailty development at early time points among high-risk individuals in many clinical settings or care institutions.

Indeed, self-reported frailty screening tools are very simple, inexpensive and practical, especially for screening frail elderly individuals in the community. However, the ideal frailty screening tool should evaluate frailty based on data that are routinely collected. Routine physical assessment and laboratory test data are the most frequently collected parameters in clinical care; for example, admission is typically related to a large number of blood tests and routine physical measures that require minimal involvement by patients. In this case, the FI-lab may be a quick and simplified assessment measure for frailty, which may contribute to a better understanding of the process from frailty to subclinical levels.

A recent proposal suggested that clinical frailty composed of clinically visible deficits represents the accumulation of subcellular, tissue and organ deficits [29, 30], and the structural and functional damages occurring at those invisible levels will eventually become irreversible and uncorrectable states [29, 31]. In addition, there is increasing evidence to suggest (at the basic molecular level) that a variety of biomarkers potentially play a role in frailty mechanisms [32–35]. These biomarkers mainly include metabolism markers, aging biomarkers and inflammatory markers (e.g., increased levels of C-reactive protein, inflammatory cytokines and glucose, as well as low levels of hemoglobin, testosterone and vitamin D). In a sense, these studies supported the earlier FI-lab findings that laboratory test abnormalities represent preclinical frailty related to microscopic deficits [36].

In our studies, the addition of laboratory test data may be helpful in understanding the associations between specific laboratory test abnormalities and frailty. Moreover, the FI-lab could serve as the basis for the early screening of older persons for frailty and risks of frailty at the cellular/molecular level. Furthermore, many routine blood tests are available in the heath care center; as a consequence, the creation of a FI-Lab using routine blood tests could be a more convenient and efficient choice for refining risk estimates for older persons and could also be integrated into the comprehensive care for those individuals, as has recently been shown by the electronic FI, which quantifies a frailty score by using routinely available, primary care health records.

In conclusion, our findings confirm the value of the FI-lab in stratifying older adults for a high risk of mortality. The feasibility and utility of adding a large number of laboratory test items to a FI may optimally improve routine frailty assessment in clinical settings; further interventions aimed at causative factors may also help in preventing the conversion of subclinical frailty into clinical frailty. For example, FI-lab should be successively applied to clinical settings to achieve modifiable intervention outcomes; thus, it may be an efficient method of informing decision-making for the better care of older patients. These considerations will guide future research by our group. Moreover, future research should investigate the association between frailty (as evaluated by the FI-Lab) and health outcomes in a clinical setting.

## Supplementary Information

**Additional file 1: Supplementary table 1.** Variables and coding used to construct the 30-item FI. **Supplementary table 2.** Baseline demographic characteristics and mortality by grades of frailty in the subjects aged over 65 years. **Supplementary table 3.** Comparison of FI-self-report vs FI-lab vs FI-combined for Prediction of 5-year mortality in the subjects aged over 65 years. **Supplementary table 4.** Baseline characteristics of the survived and dropped-out at 5 years. **Supplementary fig 1.** The flowchart of participants through the study. **Supplementary fig 2.** Receiver operating characteristic (ROC) curves.

**Additional file 2.**

## Data Availability

The datasets used and/or analyzed during the current study are available in a supplementary file.
