# Supplementary material for: A frailty index based on routine laboratory data predicts increased risk of mortality in Chinese community-dwelling adults aged over 55 years: a five-year prospective study
Source: BMC Geriatr. 2022 Aug 17;22:679. doi: 10.1186/s12877-022-03374-z (PMC9382755; doi:10.1186/s12877-022-03374-z)
Supplement: Supplementary file 1 — Additional file 1: Supplementary table 1. Variables and coding used to construct the 30-item FI. Supplementary table 2. Baseline demographic characteristics and mortality by grades of frailty in the subjects aged over 65 years. Supplementary table 3. Comparison of FI-self-report vs FI-lab vs FI-combined for Prediction of 5-year mortality in the subjects aged over 65 years. Supplementary table 4. Baseline characteristics of the survived and dropped-out at 5 years. Supplementary fig 1. The flowchart of participants through the study. Supplementary fig 2. Receiver operating characteristic (ROC) curves. [file 12877_2022_3374_MOESM1_ESM.docx]

Supplementary table 1. variables and coding used to construct the 30-item FI

| Instrument | Variable | Cut Point |
| --- | --- | --- |
| IADL disabilities | Need help with cooking meals | Limited =1; limited a little = 0.5; not limited = 0 |
|  | Need help with managing your money |  |
|  | Need help with taking a bus |  |
|  | Need help with Shopping |  |
|  | Need help with walking 300 Meters |  |
|  | Need help with up-down stairs |  |
| Psychological problems | Don’t like to make friends | Yes = 1; no = 0 |
|  | Don't adapt to change |  |
| GDS score | The geriatric depression scale | Score ≥11= 1, <11=0 |
| Self-reported morbidities | Hypertension | Yes = 1; no = 0 |
|  | Diabetes |  |
|  | Angina |  |
|  | Arrhythmia |  |
|  | Tracheitis |  |
|  | Asthma |  |
|  | Arthritis |  |
|  | Glaucoma |  |
|  | Cataract |  |
| Symptoms  Symptoms | Chronic cough | Yes = 1; no = 0 |
|  | Constipation |  |
|  | Tremor |  |
|  | Have falls |  |
|  | Have broken or fractured bones |  |
|  | Don’t hear clearly | Limited =1; limited a little = 0.5; not limited = 0 |
| MMSE score | Mini-mental State Examination Score | Score <15=1; 15-23 = 0.5; score >23 = 0 |
| Physical Performance Test  Romberg Test | Timed Up and Go Test(TUGT) | Limited =1; limited a little = 0.5; not limited = 0 |
| Romberg Test | Time to stand with the feet together and the eyes open | Time ≥ 10s = 0, < 10s = 1 |
|  | Time to stand with the open half both feet and the eyes open |  |
|  | Time to stand with the feet together and the eyes closed |  |
|  | Time to stand on two feet before and after and the eyes open |  |

IADL: Instrumental Activities Daily Living. GDS: The Geriatric Depression Scale. MMSE: Mini-Mental Status Examination.

Supplementary table 2. Baseline demographic characteristics and mortality by grades of frailty in the subjects aged over 65 years

| **Baseline Characteristic** | **Total**  **N=(843)** |  | **Grades of the FI** | | | | | | | | |
| --- | --- | --- | --- | --- | --- | --- | --- | --- | --- | --- | --- |
|  |  | **<0.20** | **0.20-0.29** | | **0.30-0.39** | | **>0.40** | | | |  |
| **FI-self-report** |  | n=672 | n=101 | | n=36 | | n=34 | | | | |
| Mean age, years (±SD) | 73.7±5.7 | 72.9±5.2 | | 75.2±5.9 |  | 77.2±5.5 | | | 80.6±6.6 | | |
| Mean FI-self-report (±SD) | 0.13±0.11 | 0.08±0.05 |  | 0.24±0.03 |  | 0.35±0.03 | |  | | 0.49±0.07 | |
| Women, n (%) | 465(55.2) | 345(51.3) |  | 73(72.3) |  | 22(61.1) | |  | | 25(73.5) | |
| Rural dwelling, n (%) | 523(62.0) | 684(64.1) |  | 88(75.9) |  | 32(86.5) | |  | | 30 (81.1) | |
| 9^+^ years education, n (%) | 236(27.9) | 215(32.4) |  | 15(15.0) |  | 4(11.1) | |  | | 2(5.9) | |
| Smoking, n (%) | 242(28.7) | 203(30.2) |  | 24(23.8) |  | 10(27.8) | |  | | 5(15.2) | |
| Drinking, n (%) | 243(28.8) | 205(30.6) |  | 23(22.8) |  | 11(30.6) | |  | | 4(12.1) | |
| Mortality, n (%) | 141(16.7) | 79(11.8) |  | 22(21.8) |  | 19(52.8) | |  | | 21(61.8) | |
| **FI-lab** |  | **<0.20** |  | **0.20-0.29** |  | **0.30-0.39** | |  | | **>0.40** | |
|  |  | n=103 |  | n=236 |  | n=153 | |  | | n=351 | |
| Mean age, years (±SD) | 73.7±5.7 | 72.3±5.3 |  | 73.4±5.8 |  | 74.1±5.7 | |  | | 74.3±5.5 | |
| Mean FI-lab (±SD) | 0.35±0.15 | 0.10±0.04 |  | 0.24±0.03 |  | 0.34±0.01 | |  | | 0.49±0.08 | |
| Women, n (%) | 465(55.2) | 49(48.0) |  | 140(59.3) |  | 81 (52.9) | |  | | 195(55.6) | |
| Rural dwelling, n (%) | 523(62.0) | 130(65.3) |  | 241(66.6) |  | 151(68.3) | |  | | 312(65.7) | |
| 9^+^ years education, n (%) | 236(27.9) | 43(41.7) |  | 68(29.3) |  | 41(27.2) | |  | | 84(24.1) | |
| Smoking, n (%) | 242(28.7) | 28(27.5) |  | 64 (27.2) |  | 42(27.5) | |  | | 107(30.5) | |
| Drinking, n (%) | 243(28.8) | 28(27.5) |  | 67 (28.5) |  | 40(26.1) |  | | | 108(30.9) | |
| Mortality, n (%) | 141(16.7) | 11(10.8) |  | 32(13.6) |  | 26(17.0) |  | | | 72(20.5) | |
| **FI-combined** |  | **<0.20** |  | **0.20-0.29** |  | **0.30-0.39** |  | | | **>0.40** | |
|  |  | n=459 |  | n=249 |  | n=99 |  | | | n=36 | |
| Mean age, years (±SD) | 73.7±5.7 | 72.4±5.1 |  | 74.6±5.5 |  | 75.7±5.9 |  | | | 78.4±6.8 | |
| Mean FI-combined (±SD) | 0.21±0.10 | 0.14±0.04 |  | 0.24±0.03 |  | 0.35±0.03 |  | | | 0.47±0.05 | |
| Women, n (%) | 465(55.2) | 231(50.3) |  | 136(54.6) |  | 71(71.7) |  | | | 27(75.0) | |
| Rural dwelling, n (%) | 523(62.0) | 259(56.4) |  | 159(63.9) |  | 76(76.8) |  | | | 29(82.1) | |
| 9^+^ years education, n (%) | 236(27.9) | 164(36.2) |  | 55(22.0) |  | 14(14.4) |  | | | 3(8.3) | |
| Smoking, n (%) | 242(28.7) | 136(29.6) |  | 74(29.7) |  | 27(27.6) |  | | | 5(13.9) | |
| Drinking, n (%) | 243(28.8) | 141(30.7) |  | 71(28.6) |  | 26(26.5) |  | | | 5(13.9) | |
| Mortality, n (%) | 141(16.7) | 49(10.7) |  | 39(15.7) |  | 31(31.3) |  | | | 22(61.1) | |

Abbreviations: FI, frailty index; SD: standard deviation.

Supplementary table 3. Comparison of FI-self-report vs FI-lab vs FI-combined for Prediction of 5-year mortality in the subjects aged over 65 years

|  |  | B | SE | Wald Statistic | HR(95%CI)^a^ | AUC (95% CI) |
| --- | --- | --- | --- | --- | --- | --- |
| **FI-self-report** | |  |  |  |  | 0.75^b^(0.72-0.78) |
| Age |  | 0.08 | 0.02 | 26.07 | 1.08(1.05-1.11)* |  |
| Female |  | -0.51 | 0.18 | 8.43 | 0.60(0.43-0.85)* | |
| FI-self-report |  | 0.04 | 0.01 | 40.69 | 1.04(1.03-1.05)* | |
| **FI-lab** |  |  |  |  |  | 0.73^c^(0.70-0.76) |
| Age |  | 0.12 | 0.01 | 69.53 | 1.12(1.09-1.15)* |  |
| Female |  | -0.30 | 0.17 | 3.06 | 0.74(0.53-1.04) | |
| FI-lab |  | 0.02 | 0.01 | 6.96 | 1.02(1.01-1.03)* | |
| **FI-combined** | |  |  |  |  | 0.76^d^(0.72-0.78) |
| Age |  | 0.09 | 0.01 | 36.43 | 1.09(1.06-1.12)* |  |
| Female |  | -0.51 | 0.18 | 8.49 | 0.60(0.43-0.85)* | |
| FI-combined | | 0.05 | 0.01 | 41.65 | 1.05(1.04-1.07)* |  |
| **FI-self-report & FI-lab** | |  |  |  |  |  |
| Age | | 0.08 | 0.02 | 27.75 | 1.08(1.05-1.11)* |  |
| Female | | -0.53 | 0.18 | 9.03 | 0.59(0.42-0.83)* |  |
| FI-self-report | | 0.04 | 0.01 | 35.63 | 1.04(1.03-1.05)* |  |
| FI-lab | | 0.01 | 0.01 | 3.28 | 1.02(1.00-1.03)* |  |

Abbreviations: FI, frailty index; AUC, area under the curve; CI, confidence interval; SE: standard error; HR, hazard ratio; ^a^ Adjusted for age and sex.

^b^*p*=.097 for comparison of FI-self-report vs FI-lab.

^c^*p*=.038 for comparison of FI-lab vs FI-combined.

^d^*p*=.786 for comparison of FI-self-report vs FI-combined.

Supplementary table 4. Baseline characteristics of the survived and dropped-out at 5 years

|  | **Survived** | **Dropped out** | *p* |
| --- | --- | --- | --- |
| N | 946 | 156 |  |
| Age, years (SD) | 68.0 (7.6) | 69.3 (8.5) | 0.055 |
| Female, n (%) | 547 (57.8) | 87 (55.8) | 0.631 |
| 9^+^ year education, n (%) | 277 (29.6) | 89 (57.8) | <0.001 |
| Smoking, n (%) | 284 (30.1) | 41 (26.3) | 0.193 |
| BMI | 24.1(3.9) | 24.7 (3.8) | 0.108 |
| Hypertension, n (%) | 332 (35.1) | 47( 30.5) | 0.052 |
| Diabetes, n (%) | 85 (9.0) | 25(14.7) | 0.126 |
| ADL score | 6.1(0.8) | 6.0 (0.3) | 0.057 |

Died (n=155)

Missing (n=156)

**Year 2009(baseline)** Total of 1257(550 males and 707 females) older adults completed the face-to-face interview and physical examination

**Year 2014(5-year follow-up)**

Supplementary fig 1. The flowchart of participants through the study

Supplementary fig 2. Receiver operating characteristic (ROC) curves

ROC curves showing the performance of the frailty index on predicting 5-year mortality in subjects aged over 65 years. The area under the curve (mean AUC±standard error）were 0.73±0.02 for FI-lab within 5 years; AUC=0.75±0.02 for FI-self-report within 5 years; AUC=0.76±0.02 for FI-combined within 5 years. The black diagonal line indicates a reference area under the ROC curve (AUC) of 0.50 (no better than chance alone).
